# Conotoxins that Confer Therapeutic Possibilities 

**DOI:** 10.3390/md10061244

**Published:** 2012-06-04

**Authors:** Magbubah Essack, Vladimir B. Bajic, John A. C. Archer

**Affiliations:** Computational Bioscience Research Center (CBRC), King Abdullah University of Science and Technology (KAUST), Thuwal 23955-6900, Jeddah, Kingdom of Saudi Arabia; Email: magbubah.essack@kaust.edu.sa (M.E.); vladimir.bajic@kaust.edu.sa (V.B.B.)

**Keywords:** *Conus*, cone snail, peptide, neuropeptide, conotoxin, nicotinic acetylcholine receptor, sodium channel, calcium channel, potassium channel

## Abstract

Cone snails produce a distinctive repertoire of venom peptides that are used both as a defense mechanism and also to facilitate the immobilization and digestion of prey. These peptides target a wide variety of voltage- and ligand-gated ion channels, which make them an invaluable resource for studying the properties of these ion channels in normal and diseased states, as well as being a collection of compounds of potential pharmacological use in their own right. Examples include the United States Food and Drug Administration (FDA) approved pharmaceutical drug, Ziconotide (Prialt^®^; Elan Pharmaceuticals, Inc.) that is the synthetic equivalent of the naturally occurring ω-conotoxin MVIIA, whilst several other conotoxins are currently being used as standard research tools and screened as potential therapeutic drugs in pre-clinical or clinical trials. These developments highlight the importance of driving conotoxin-related research. A PubMed query from 1 January 2007 to 31 August 2011 combined with hand-curation of the retrieved articles allowed for the collation of 98 recently identified conotoxins with therapeutic potential which are selectively discussed in this review. Protein sequence similarity analysis tentatively assigned uncharacterized conotoxins to predicted functional classes. Furthermore, conotoxin therapeutic potential for neurodegenerative disorders (NDD) was also inferred.

## 1. Introduction

Cone Snails (genus *Conus*) are invertebrate venomous predators comprising approximately 700 species [1], with each *Conus* species producing a distinctive repertoire of 100–200 venom peptides [2]. The venom peptides are used to immobilize and digest prey as well as to defend cone snails from predators. It has been demonstrated that most *Conus* peptides potently and specifically target the voltage- and ligand-gated ion channels in the nervous systems of prey. These *Conus* peptides also act on homologous mammalian ion channels due to the degree of structural conservation exhibited by the voltage- and ligand-gated ion channels across higher eukaryotes. Moreover, mammalian ion channels exhibit diverse tissue expression patterns. This difference in tissue expression patterns was demonstrated with conotoxins that target the nicotinic acetylcholine receptor (nAChR) subtypes present at the invertebrate neuromuscular junctions which, while not present in vertebrate neuromuscular junctions, are expressed in tissues relevant to pain. Thus peptides that target these ion channels may potentially be analgesic therapeutic agents in vertebrates [3]. 

*Conus* peptides, such as the μ-conotoxins and ω-conotoxins, are currently being used as standard research tools in neuroscience. The μ-conotoxins are used for the immobilization of skeletal muscles without affecting axonal or synaptic events because of their ability to block the muscle Na^+^ channel Na_v_1.4, but not axonal Na^+^ channels Na_v_1.1–Na_v_1.3 and Na_v_1.6–Na_v_1.9 [4,5]. The ω-conotoxins are used as standard pharmacological reagents in voltage-gated calcium (Ca^2+^) channel-related research and are used to block neurotransmitter release [6,7]. ω-conotoxins have also been used to diagnose the Ca^2+^ channel targeted disease, Lambert-Eaton myasthenic syndrome [8]. Moreover, Ziconotide (Prialt^®^; Elan Pharmaceuticals, Inc.) is the first United States Food and Drug Administration (FDA) approved cone snail-derived pharmaceutical drug. Ziconotide is a synthetic equivalent of a naturally occurring conopeptide known as SNX-111 or ω-conotoxin MVIIA that was isolated from the cone snail, *Conus magus* [3]*.* This ω-conotoxin MVIIA targets the N-type Ca^2+^ channels that are related to algesia in the nervous system and is thus being used for the treatment of severe chronic pain in patients requiring intrathecal (IT) administration of drugs [9]. 

Other cone snail-derived peptides such as CGX-1007, CGX1160, CGX-1051, ACV1 and Xen2174, are now being tested in clinical trials. CGX-1007 (Conantokin G) isolated from the cone snail, *Conus geographus*, is a *N*-methyl-D-aspartate (NMDA) receptor antagonist that is being screened as a potential treatment for epileptic seizures [10]. CGX1160 (Contulakin-G) also isolated from *Conus geographus* [11], is a neurotensin subtype 1 (NTS1) receptor agonist that is being screened as a potential treatment of severe chronic pain in patients requiring IT administration of drugs [12]. CGX-1051 isolated from the cone snail, *Conus purpurasens*, is a potassium (K^+^) channel inhibitor that is being screened as a potential treatment for heart myocardial infarction [13]. ACV1 (conotoxin Vc1.1) identified from the cone snail, *Conus victoriae* is a neuronal nAChR antagonist that is in multiple trials as a potential treatment for sciatic neuropathic pain and diabetic neuropathy or post herpetic neuralgia [14]. Xen2174 (Mr1A) isolated from the cone snail, *Conus marmoreus*, is also a nAChR antagonist that is being screened as a potential treatment for chronic neuropathic [15] and post-surgical pain [16]. In addition, a plethora of *Conus* peptides have been demonstrated to: (1) induce antinociceptive [17], antiepileptic [18], neuroprotective or cardioprotective activities [19,20]; and (2) have potential relevance in cancer [21] and neuronal diseases [22,23].

In light of these encouraging reports of conotoxin-related research, here we review recently isolated *Conus* peptides that may have the potential to be developed into therapeutic drugs. We used the National Center for Biotechnology Information (NCBI) PubMed database [24] to search for cone snail derived lead compounds using the following keywords: “*Conus* OR cone snail OR conotoxin OR conopeptide”.

**Figure 1:**
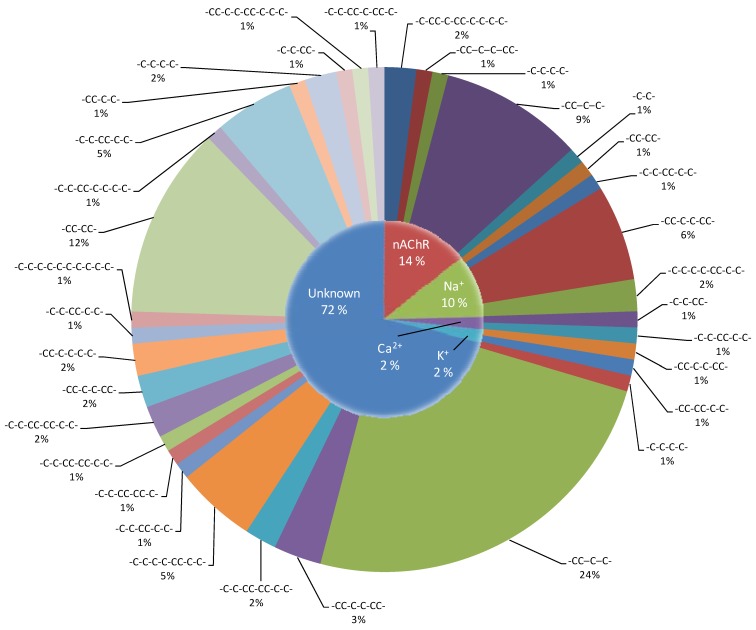
Peptides isolated from cone snails since the 1 January 2007 to 31 August 2011, categorized by their respective targets.

This query was limited to articles published from 1 January 2007 to 31 August 2011, so as to include only recently isolated *Conus* peptides. This yielded a total of 1129 documents, curation of which allowed for the identification of 98 *Conus* peptides that have potential to be used to generate new drugs. Here we present an overview of the 98 conotoxins that have been reported in literature from 1 January 2007 to 31 August 2011, correlated with the conotoxins cysteine arrangement and their known targets (Figure 1). The compounds identified constitute five phenotypic classes: (1) 14 nAChR inhibitors; (2) 10 Na^+^ channel inhibitors; (3) 2 Ca^2+^ channel inhibitors; (4) 2 K^+^ channel inhibitors; and (5) 70 peptides with targets that have not been defined (Supplementary Table S1).

## 2. *Conus* Peptides That Exhibit Therapeutic Potential

### 2.1. Voltage-Gated Ion Channels Targeted by Conotoxins

Of the conotoxins highlighted in this review, 14% (14/98) have been demonstrated to inhibit the Na^+^, Ca^2+^ or K^+^ channels. These are transmembrane proteins that mediate the excitability of nerve and muscle cells. To date, nine mammalian Na^+^ channel α subunits (Na_v_1.1–Na_v_1.9) have been identified and characterized with respect to sensitivity to the neurotoxin tetrodotoxin (TTX) [5]. These Na^+^ channels are modulated by numerous natural toxins, either by blocking current through the pore or by modifying channel gating [25]. Na^+^ channel subtypes Na_v_1.8 and Na_v_1.9 have been characterized as being tetrodotoxin-resistant (TTX-R) and are implicated in neuropathic pain states [26]. On the other hand, Na^+^ channel subtypes Na_v_1.7, Na_v_1.3, Na_v_1.2 and Na_v_1.1 are tetrodotoxin-sensitive (TTX-S) and implicated in neuropathic pain [27], inflammation [26] and epilepsy [28,29]. Similarly, more than 40 known human K^+^ channel α subunits have been identified and implicated in numerous disorders [30]. Some examples are: (1) K_v_7 has been implicated in cerebral vasospasm [31]; (2) K_v_1.4 has been implicated in trigeminal inflammatory allodynia in temporomandibular joint (TMJ) disorder [32]; (3) K_v_1.2, K_v_1.3 and K_v_1.6 have been shown to be key regulators in Dopamine release, the dysfunction of which is thought to be implicated in drug abuse and in diseases such as schizophrenia and Parkinson’s disease [33]; (4) K_v_1.3 has also been shown to a target for immunosuppression [34]; (5) K_v_2.1 has been implicated in hypoxia/anoxia induced cell apoptosis [35] and diabetes [36] and; (6) mutations in K_v_1.1 have been implicated in autosomal dominant hypomagnesemia and episodic ataxia type 1 [37]. Ten Ca^2+^ channel subtypes have also been identified (Ca_v_1.1, Ca_v_1.2, Ca_v_1.3, Ca_v_1.4, Ca_v_2.1, Ca_v_2.2, Ca_v_2.3, Ca_v_3.1, Ca_v_3.2 and Ca_v_3.3) and implicated in numerous disorders too. Some examples are: (1) Ca_v_2.1 (P/Q type) and Ca_v_2.2 (N-type) have been implicated in bladder nociception [38]; (2) Ca_v_1.3 (L-type) has been implicated in Parkinson’s disease [39]; (3) Ca_v_3.1, Ca_v_3.2 and Ca_v_3.3 (T-type) have been implicated in age-related neurodegenerative disorders [40]; and (4) Ca_v_2.3 (R-type) has been implicated in diabetes [41]. To understand the function of voltage-gated ion channel subtypes in the normal and disease states will require novel inhibitors with improved voltage-gated ion channel subtype selectivity.

#### 2.1.1. Na^+^ Channel Inhibitors

Lt5d: Liu *et al.* (2007) isolated the novel conotoxin, Lt5d, from the venom of *Conus litteratus* [42]. Lt5d was identified as a T-1-conotoxin comprising 12 amino acid residues with a characteristic arrangement of four-cysteine residues (-CC-CC-) (Table 1). It was further demonstrated that Lt5d inhibit tetrodotoxin-sensitive (TTX-S) sodium currents on adult rat dorsal root ganglion (DRG) neurons (IC_50_ 156.16 nM), but has no effect on tetrodotoxin-resistant (TTX-R) sodium currents treated with 150 nM Lt5d [42]. Thus, Lt5d is the first T-1-conotoxin shown to inhibit TTX-S Na^+^ channels. 

Lt6c: Wang *et al.* (2008) isolated Lt6c from the venom of *Conus litteratus* as well [43]. Lt6c was shown to comprise 28 amino acid residues with a characteristic arrangement of the six-cysteine residues (-C-C-CC-C-C-) (Table 1). It was further demonstrated that 800 nM Lt6c inhibits both the TTX-S and TTX-R sodium currents on adult rat DRG neurons [43].

**Table 1:** Amino acid sequence and conserved cysteine residues of the recently identified Na^+^ channel targeting conotoxins.

Peptide	AA Sequence	Gene Family with Cysteine Framework and Residues	Targets	Has no Effect on	Reference
Lt5d	DCCPAKLLCCNP	T superfamily V [connectivity I–III, II–IV] -CC-CC-	Na^+^ channel	ND	[42]
Lt6c	WPCKVAGSPCGLVSECCGTCNVLRNRCV	O1 superfamily VI/VII [connectivity I–IV, II–V, III–VI] -C-C-CC-C-C-	Na^+^ channel	ND	[43]
TIIIA	RHGCCKGOKGCSSRECRPQHCC	M superfamily III -CC-C-C-CC-	rNa_v_1.2 rNa_v_1.4	rNa_v_1.3 rNa_v_1.5 rNa_v_1.7 rNa_v_1.8	[44]
Cal12a	DVCDSLVGGHCIHNGCWCDQEAPHGNCCDTDGCTAAWWCPGTKWD	O2 superfamily XII -C-C-C-C-CC-C-C-	Na^+^ channel	ND	[45]
Cal12b	DVCDSLVGGHCIHNGCWCDQDAPHGNCCDTDGCTAAWWCPGTKWD	O2 superfamily XII -C-C-C-C-CC-C-C-	Na^+^ channel	ND	[45]
BuIIIA	VTDRCCKGKRECGRWCRDHSRCC	M superfamily III -CC-C-C-CC-	Na_v_1.4	ND	[46]
BuIIIB	VGERCCKNGKRGCGRWCRDHSRCC	M superfamily III -CC-C-C-CC-	Na_v_1.4	ND	[46]
BuIIIC	IVDRCCNKGNGKRGCSRWCRDHSRCC	M superfamily III -CC-C-C-CC-	Na_v_1.4	ND	[46]
SIIIA	ZNCCNGGCSSKWCRDHARCC	M superfamily III -CC-C-C-CC-	rNa_v_1.2 rNa_v_1.4	rNa_v_1.3 rNa_v_1.5 rNa_v_1.7 rNa_v_1.8	[47]
SIIIB	ZNCCNGGCSSKWCKGHARCC	M superfamily III -CC-C-C-CC-	rNa_v_1.2 rNa_v_1.4	rNa_v_1.3 rNa_v_1.5 rNa_v_1.7 rNa_v_1.8	[47]

ND = no data.

TIIIA: Lewis *et al.* (2007) isolated the novel conotoxin, TIIIA, from the venom of *Conus tulipa* [44]. TIIIA was identified as a μ-conotoxin comprising 22 amino acid residues with a characteristic arrangement of the six-cysteine residues (-CC-C-C-CC-) (Table 1). TIIIA was further demonstrated to inhibit Na^+^ channel subtype rNa_v_1.2 (IC_50_ of 40 nM) and rNa_v_1.4 (IC_50_ of 9 nM). Moreover, no effect was demonstrated on the Na^+^ channel subtypes rNa_v_1.3, rNa_v_1.5, rNa_v_1.7 and rNa_v_1.8 induced with 3 μM TIIIA. Also, the TIIIA analog [E15A]TIIIA (IC_50_ of 15 pM) had a 10-fold higher affinity than TIIIA (IC_50_ of 148 pM) for TTX-S Na^+^ channels [44]. 

Cal12a and Cal12b: Gilly* et al.* (2011) isolated two novel conotoxins, Cal12a and Cal12b, from the venom of *Conus Californicus* [45]. Both Cal12a and Cal12b were identified as μ-conotoxins comprising 45 amino acid residues with eight-cysteine residues in framework 12 (-C-C-C-C-CC-C-C-) (Table 1). It was further demonstrated that Cal12a and Cal12b reversibly block the Na^+^ channels on giant-fiber-lobe (GFL) neurons, but have no effect on Ca^2+^ and K^+^ channels [45]. 

BuIIIA, BuIIIB and BuIIIC: Holford* et al.* (2009) identified the novel conotoxins BuIIIA, BuIIIB and BuIIIC, by cDNA cloning and peptide purification from* Conus bullatus*. BuIIIA, BuIIIB and BuIIIC were also identified as μ-conotoxins have a characteristic arrangement of six-cysteine residues (-CC-C-C-CC-) and comprising 23, 24 and 26 *amino acid* residues, respectively (Table 1). Activities of these compounds were compared to a representative set of μ-conotoxins, PIIIA, GIIIA, and KIIIA. BuIIIA and KIIIA were demonstrated to reversibly block the Na^+^ channel skeletal muscle subtype Na_v_1.4 with similar potency. In contrast, BuIIIB and BuIIIC were demonstrated to be more potent irreversible inhibitors of the Na^+^ channel subtype Na_v_1.4, similar to the reversible inhibitors of Na_v_1.4, PIIIA and GIIIA [46]. The novel structural determinants of BuIIIA, BuIIIB, and BuIIIC along with their ability to potently inhibit Na_v_1.4 make these conotoxins useful in defining features of the Na_v_1.4 pharmacophore and thereby facilitate the design of highly subtype-specific ligands that target Na_v_1.4.

SIIIA and SIIIB: Schroeder* et al.* (2008) isolated the novel conotoxins, SIIIA and SIIIB, from the venom of *Conus striatus* [47]. Both SIIIA and SIIIB were identified as μ-conotoxin comprising 20 amino acid residues with a characteristic arrangement of six-cysteine residues (-CC-C-C-CC-) (Table 1). SIIIB was further demonstrated to inhibit Na^+^ channel subtype rNa_v_1.2 (IC_50_ of 5 nM) and rNa_v_1.4 (IC_50_ of 3 nM) more potently than SIIIA (rNa_v_1.2: IC_50_ of 10 nM and rNa_v_1.4: IC_50_ of 60 nM). However, SIIIA is the more selective ligand for rNa_v_1.2 as it has a high potency for rNa_v_1.2 and also shows a larger difference in IC_50_ between rNa_v_1.2 and rNa_v_1.4. Furthermore, *Xenopus* oocytes treated with 3 μM SIIIA and SIIIB showed little to no effect on Na^+^ channel subtypes Na_v_1.3, Na_v_1.5, Na_v_1.7 and Na_v_1.8 [47]. 

#### 2.1.2. Ca^2+ ^Channel Inhibitors

FVIA: Lee et al.* (2010)* identified the novel conotoxin, FVIA, by cDNA cloning and peptide purification from *Conus fulmen*. FVIA was identified as a ω-conotoxin comprising 25 amino acid residues with a characteristic arrangement of six-cysteine residues (-C-C-CC-C-C-) (Table 2). FVIA activity was compared to the known Ca^2+^ channel inhibitor, MVIIA. Both FVIA (IC_50_ of 11.5 nM) and MVIIA (IC_50_ of 7.96 nM) were shown to inhibit human N-type Ca^2+^ channels stably expressed in HEK293 cells (C2D7 cells), but FVIA shows greater reversibility than MVIIA. FVIA was further demonstrated to have no effect on other Ca^2+^ channels (T-type and P/Q-type) and TTX-sensitive Na^+^ channels of mouse DRG neurons [48].

CalTx: Bernaldez *et al.* (2011) isolated novel conotoxin, CalTx, from the venom of *Conus californicus* as well [49]. CalTx was shown to comprise 13 amino acid residues with a characteristic arrangement of four-cysteine residues (-C-C-CC-) (Table 2). In contrast to Cal12a and Cal12b that show no effect on Ca^2+^ channels, CalTx was further demonstrated to reversibly block calcium current in rat DRG neurons treated with 20 μM CalTx [49].

**Table 2:** Amino acid sequence and conserved cysteine residues of the recently identified Ca^2+^ channel targeting conotoxins.

Peptide	AA Sequence	Gene Family with Cysteine Framework and Residues	Targets	Has no Effect on	Reference
CalTx	NCPAGCRSQGCCM	XVI -C-C-CC-	N-type L-type P/Q-type R-type	T-type	[49]
FVIA	CKGTGKSCSRIAYNCCTGSCRSGKC	O1 superfamily VI/VII [connectivity I–IV, II–V, III–VI] -C-C-CC-C-C-	N-type	T-type P/Q-type TTX-S Na^+^ channel	[48]

#### 2.1.3. K^+^ Channel Inhibitors

Sr11a: Aguilar *et al.* (2007) isolated the novel conotoxin, Sr11a, from the venom of *Conus spurius*. Sr11a was identified as a I-conotoxin comprising 22 amino acid residues with a characteristic arrangement of six-cysteine residues (-CC-CC-C-C-) (Table 3) [50]. In 2010, it was further demonstrated that Sr11a inhibits the K^+^ channel subtype K_v_1.2 (IC_50_ of 66 nM) and K_v_1.6 (IC_50_ of 58 nM), but shows no effect on K_v_1.3 treated with up to 10 Mm Sr11a [51].

**Table 3:** Amino acid sequence and conserved cysteine residues of the recently identified K^+^ channel targeting conotoxins.

Peptide	AA Sequence	Gene Family with Cysteine Framework and Residues	Targets	Has no Effect on	Reference
Sr11a	NQQCCWRSCCRGECEAPCRFGP	I2 superfamily XI [connectivity I–IV, II–VI, III–VII, V–VIII] -CC-CC-C-C-	K_v_1.2 K_v_1.6	K_v_1.3	[50,51]
RIIIj	LPPCCTPPKKHCPAPACKYKPCCKS	M superfamily III -CC-C-C-CC-	K_v_1.2	K_v_1.1 K_v_1.3 K_v_1.4 K_v_1.5 K_v_1.6 KCNQ2/KCNQ3 BK	[20]

RIIIJ: Chen *et al.* (2010) isolated the novel conotoxin, RIIIJ, from the venom of *Conus radiatus*. RIIIJ was identified as a κM-conotoxin comprising 25 amino acid residues with a characteristic arrangement of six-cysteine residues (-CC-C-C-CC-) (Table 3). The activity of this compound was compared to the known K^+^ channel inhibitor, RIIIK. RIIIJ (IC_50_ of 33 nM) was shown to reversibly inhibit the K^+^ channel subtype K_v_1.2 with a higher potency than RIIIK (IC_50_ of 352 nM). Both RIIIJ and RIIIK showed very low or no affinity for K^+^ channel subtype K_v_1.1, K_v_1.3, K_v_1.4, K_v_1.5, K_v_1.6, KCNQ2/KCNQ3 and BK [20].

### 2.2. Ligand-Gated Ion Channels Targeted by Conotoxins

This review additionally highlights that 14% (14/98) of the novel identified conotoxins are also nAChR inhibitors. nAChR respond to endogenous agonists including acetylcholine and choline and participate in an extensive range of processes including cognitive function, motor movement, sound perception and immune function. nAChRs are allosteric transmembrane proteins composed of one or more α subunits (α1–α10) either alone or in combination with one or more non-α-subunits, (β subunits (β1–β4), γ, δ or ε), that together make up the functional ligand-gated ion channel complex; all nAChRs are believed to contain five such subunits. nAChR subtypes show distinct anatomical location, unique biophysical and pharmacological properties, and additionally have been implicated in numerous disorders. Some examples are: (1) α6β2 and α4β2 has been implicated in Parkinson’s disease [52], (2) α7 has been implicated in Alzheimer’s disease [53] and schizophrenia [54] and has been identified as the target for chemotherapy-related cognitive impairment [55]; and (3) α9α10 has been identified as the target for the development of analgesics for the treatment of chronic neuropathic pain [56]. To understand the functioning of these ligand-gated ion channel subtypes in the normal and disease states requires novel inhibitors with improved ligand-gated ion channel subtype selectivity.

#### nAChR Inhibitors

AlphaD-cap (αD-cap) and AlphaD-mus (αD-mus): Kauferstein *et al.* (2009) isolated two novel conopeptides, αD-Cp and αD-Ms, from the venom of *Conus capitaneus* and *Conus mustelinus*, respectively [57]. Both αD-Cp and αD-Ms were shown to be structurally homologous to the αD-conopeptides (αD-VxXIIA, -B and -C) isolated from the venom of *Conus vexillum* [58], comprising 49 *amino acid* residues and having a characteristic arrangement of ten-cysteine residues (-C-CC-C-CC-C-C_9_C-C-) (Table 4). αD-Cp and αD-Ms were further demonstrated to specifically block neuronal nicotinic acetylcholine receptors (nAChRs). αD-Cp showed the same selectivity profile for the nAChR subtypes as αD-Ms, but has a lower potency. αD-Ms demonstrated selectivity for the α7 (IC_50_ 0.12 nM), α3β2 (IC_50_ 1.08 nM) and α4β2 (IC_50_ 4.5 nM) neuronal nAChR subtypes. Both peptides showed no effect on the nAChR subtypes α3β4 and α4β4 and the muscle nAChR subtype α1β1γδ at concentrations up to 3 μM [57].

αC-PrXA: Jimenez* et al.* (2007) isolated the novel conotoxin, αC-PrXA, from the venom of *Conus parius* [59]. αC-PrXA is an unusual αC-conotoxin (-C-C-) comprising 32 amino acid residues (Table 4) and it is most similar in its biochemical features to snake toxin Waglerins. This compound was further demonstrated to potently block the skeletal muscle nAChR subtypes α1β1γδ (IC_50_ 3.0 nM) and α1β1εδ (IC_50_ 1.8 nM). Moreover, little to no effect was demonstrated on neuronal nAChRs (subtypes α7, α3β2, α3β4, α2β4, α4β2 and α9α10), NMDA receptors (subtypes NR2A and NR2B) and Na^+^ channels (subtypes Na_v_1.4 and Na_v_1.6) induced with 10 μM αC-PrXA [59]. 

PrIIIE: Lluisma *et al.* (2009) identified the novel conotoxin, PrIIIE, by cDNA cloning and peptide purification from *Conus parius* as well. PrIIIE was identified as a ψ-conotoxin comprising 24 amino acid residues (Table 4) with a characteristic arrangement of six-cysteine residues (-CC-C-C-CC-) [60], similar to ψ-conotoxins, PIIIE and PIIIF, previously isolated from *Conus purpurascens* [61]. It was further demonstrated that PrIIIE blocks the nAChR subtypes α1β1γδ (IC_50_ 0.25 μM) and α1β1εδ (IC_50_ 3.24 μM). Unlike PIIIE, PrIIIE demonstrated no effect on the Na^+^ channel subtype Nav1.4 induced with 5 nM PrIIIE. Moreover, PrIIIE (IC_50_ 0.25 μM) was shown to be a significantly more potent nAChR receptor inhibitor than PIIIE (IC_50_ 7 μM) [60].

Pu14a and Ts14a: Peng *et al.* (2010) identified two novel conotoxins, Pu14a and Ts14a, by cDNA cloning and peptide purification from *Conus pulicarius and Conus tessulatus*, respectively. Both Pu14a and Ts14a contain the characteristic arrangement of four separate cysteine residues (C-C-C-C) and additionally share high sequence similarity comprising 19 amino acid residues (Table 4). However, only Pu14a was further demonstrated to block the nAChR subtypes α3β2 (IC_50_ 10 μM), α6α3β2 (IC_50_ 1 μM) and α1β1γδ (IC_50_ 1 μM). Moreover, Pu14a showed no effect on the K^+^ channels in mouse superior cervical ganglion neurons [62].

α-PIB: Lopez-Vera *et al.* (2007a) isolated the novel conotoxin, α-PIB, from the venom of *Conus purpurascens* [63]. α-PIB is an unusual α4/4-conotoxin (-CC-C-C-) comprising 16 amino acid residues (Table 4). This compound was further demonstrated to specifically block the skeletal muscle nAChR subtypes α1β1γδ (IC_50_ 45 nM) and α1β1εδ (IC_50_ 36 nM). Moreover, no effect was demonstrated on nAChR subtypes α7, α3β4, α3β2, α2β4 and α9α10 induced with 10 μM α-PIB [63]. 

SrIA and SrIB: Lopez-Vera *et al.* (2007b) isolated two novel α-conotoxins, SrIA and SrIB, from the venom of *Conus spurius* and synthesized the synthetic analog [γ15E]SrIB, by substituting glutamate for the γ-carboxyglutamate residue [64]. Both peptides along with [γ15E]SrIB comprise 18 amino acid residues with the typical 4/7-type framework (-CC-C-C-) (Table 4) and thus were compared to the α4/7-conotoxin EI previously isolated from the from *Conus ermineus* [65]. The results with [γ15E]SrIB were shown not to be significantly different from the natural compounds, thus [γ15E]SrIB was used for further testing owing to the limited availability of the natural toxins SrIA and SrIB. EI demonstrated strong blocking of the nAChR subtypes α4β2, α1β1γδ and α3β4 at 10 μΜ, whilst the novel peptides only demonstrated weak blocking of the nAChR subtypes α4β2 and α1β1γδ at the same concentration. However, SrIA, SrIB and [γ15E]SrIB induces strong potentiation of the nAChR subtypes α4β2 and α1β1γδ (IC_50_ 1.78 nM) [64]. 

Ac1.1a and Ac1.1b: Yuan *et al.* (2007) identified novel conotoxins, Ac1.1a and Ac1.1b, by cDNA cloning and peptide purification from *Conus* *achatinus*. It was shown that Ac1.1a and Ac1.1b are α3/5 conotoxins comprising 17 amino acid residues with a characteristic arrangement of four-cysteine residues (-CC-C-C-) (Table 4) [66]. Liu* et al.* (2007) further demonstrated that both Ac1.1a and Ac1.1b block the nAChR subtypes α1β1γδ (Ac1.1a: IC_50_ 35.90 nM; Ac1.1b: IC_50_ 25.80 nM), α1β1εδ (Ac1.1a: IC_50_ 3.20 nM; Ac1.1b: IC_50_ 0.10 nM), α2β2 (Ac1.1a and Ac1.1b: IC_50_ > 5000 nM), α3β4 (Ac1.1a and Ac1.1b: IC_50_ > 50,000 nM) and α1γβ1 (Ac1.1a and Ac1.1b: IC_50_ > 50,000 nM), indicating that both toxins strongly prefer the α1-δ subunit interface instead of the α1-γ binding site on the nAChRs [67].

ArIA and ArIB: Whiteaker *et al.* (2007) identified the novel conotoxins, ArIA and ArIB, by cDNA cloning and peptide purification from *Conus arenatus.* It was shown that both ArIA and ArIB have the characteristic arrangement of four-cysteine residues (-CC-C-C-) and are α4/7 conotoxins comprising 22 and 20 amino acid residues, respectively (Table 4) [68]. These compounds were further demonstrated to specifically block the nAChR subtypes α7 and α3β2. ArIB (IC_50_ 1.81 nM) blocked α7 more potently than ArIA (IC_50_ 6.02 nM), whilst ArIA (IC_50_ 18.0 nM) blocked α3β2 more potently than ArIB (IC_50_ 60.1 nM) [68]. Taken together, ArIB is the more selective ligand for α7 nAChRs as it has a higher potency for α7 and also showed a larger difference in IC_50_ between α7 and α3β2 nAChRs.

α-TxIA and TxIA(A10L): Dutertre *et al.* (2007) isolated the novel α-conotoxin, α-TxIA, from the venom of *Conus textile* and synthesized its synthetic analog TxIA(A10L) [69]. Both α-TxIA and its synthetic analog TxIA(A10L) comprise 16 amino acid residues with a characteristic arrangement of four-cysteine residues (-CC-C-C-) (Table 4). These compounds were further demonstrated to block the neuronal nAChR subtypes α7 and α3β2. The α3β2 nAChR was selectively targeted by both α-TxIA (IC_50_ 3.6 nM) and TxIA(A10L) (IC_50_ 2.0 nM), whilst TxIA(A10L) (IC_50_ 39 nM) blocks the α7 nAChR tenfold more potently than α-TxIA (IC_50_ 392 nM). Moreover, both compounds exerted no effect on the α4β2 nAChR and muscle nAChR at concentrations up to 10 μM [69].

**Table 4:** Amino acid sequence and conserved cysteine residues of the recently identified nAChR targeting conotoxins.

Peptide	AA Sequence	Gene Family with Cysteine Framework and Residues	nAChR Targets	Has no Effect on	Reference
AlphaD-cap	EVQECQVDTPGSSWGKCCMTRMCGTMCCSRSVCTCVYHWRRGHGCSCPG	D superfamily XX -C-CC-C-CC-C-C-C-C-	α7 α3β2 α4β2	α3β4 α4β4	[57]
AlphaD-mus	DVRECQVNTPGSKWGKCCMTRMCGTMCCARSGCTCVYHWRRGHGCSCPG	D superfamily XX -C-CC-C-CC-C-C-C-C-	α7 α3β2 α4β2	α3β4 α4β4	[57]
α-PIB	ZSOGCCWNPACVKNRC	A superfamily I [connectivity I–III, II–IV] -CC-C-C-	α1β1εδ α1β1γδ	α7 α3β4 α3β2 α2β4 α9α10	[63]
SrIA	RTCCSROTCRMγYPγLCG	A superfamily I [connectivity I–III, II–IV] -CC-C-C-	α4β2 α1β1γδ	α3β4	[64]
SrIB	RTCCSROTCRMEYPγLCG	A superfamily I [connectivity I-III, II-IV] -CC-C-C-	α4β2 α1β1γδ	α3β4	[64]
Pu14a	DCPPHPVPGMHKCVCLKTC	A superfamily XIV [connectivity I–III, II–IV] -C-C-C-C-	α3β2 α6α3β2 α1β1γδ	K^+^ channels	[62]
PrIIIE	AARCCTYHGSCLKEKCRRKYCCGR	M superfamily III -CC-C-C-CC-	α1β1εδ α1β1γδ	Na_v_1.4	[60]
ArIA	IRDECCSNPACRVNNOHVCRRR	A superfamily I [connectivity I-III, II-IV] -CC-C-C-	α7 α3β2	ND	[68]
ArIB	DECCSNPACRVNNPHVCRRR	A superfamily I [connectivity I-III, II-IV] -CC-C-C-	α7 α3β2	ND	[68]
Ac1.1a	NGRCCHPACGKHFNCGR	A superfamily I [connectivity I-III, II-IV] -CC-C-C-	α1β1γδ α1β1εδ α2β2 α3β4 α1γβ1	ND	[66,67]
Ac1.1b	NGRCCHPACGKHFNCGR	A superfamily I [connectivity I-III, II-IV] -CC-C-C-	α1β1εδ α1β1γδ α2β2 α3β4 α1γβ1	ND	[66,67]
PrXA	TYGIYDAKPOFSCAGLRGGCVLPONLROKFKE	-C-C-	α1β1εδ α1β1γδ	α7 α3β2 α3β4 α2β4 α4β2 α9 α10 Na_v_1.4 Na_v_1.6 NR2A NR2B	[59]
α-TxIA	GCCSRPPCIANNPDLC	A superfamily I [connectivity I-III, II-IV] -CC-C-C-	α7 α3β2	α4β2 α1β1εδ α1β1γδ	[69]
TxIA(A10L)	GCCSRPPCILNNPDLC	A superfamily I [connectivity I-III, II-IV] -CC-C-C-	α7 α3β2	α4β2 α1β1εδ α1β1γδ	[69]

ND = no data.

## 3. Prediction of Conotoxin Targets

By determining their protein sequence similarities, and potential number of disulfide bridges and the types of cysteine arrangement of conotoxins with known targets to the 70 conotoxins with undetermined targets using Blastp [70], we predicted the targets of the conotoxins whose targets are currently not defined (Supplementary Table S1). Multiple alignments of Blastp results discussed in this review are presented using ClustalW2 [71]. Thus conotoxin targets are inferred based on sequence similarity. Targets could not be predicted for all conotoxins as some conotoxins such as Ca11a (I3 superfamily), Ca11b (I3 superfamily), Ca8a (S superfamily), Vi15a (V superfamily) and Ca16a (Y superfamily) belong to newly defined gene superfamilies, whilst others such as Mr1e have not been assigned to a gene superfamily as yet. Also, I2 superfamily conotoxins such as Eb12.4, Im12.10, Mr12.5, Mr12.8, Lt12.4, Lt12.9 and TxX sequence similarity infer that they may have a similar target but there are currently no known targets for conotoxins of this type (Supplementary Figure S1). Similarly, conotoxins Pr3a, Ar11a, Qc16a, Pu5.2*,* Sr7a, Pr6a, Pr6b, Pr6c, Pr6d and Pu5.3 also show no significant sequence similarity to conotoxins with known targets. 

**Figure 2:**
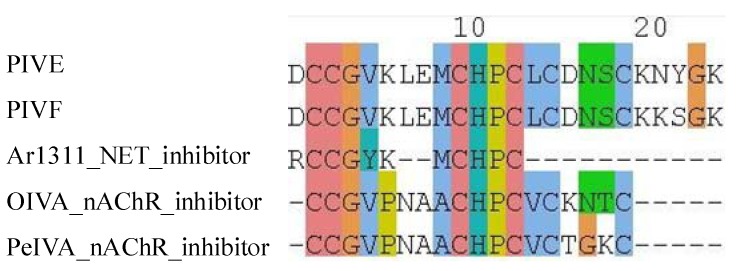
Multiple alignment of A superfamily conotoxins belonging to cysteine framework IV predicted to target nAChR.

However, in a sequence similarity search (Supplementary Figure S1) all the A superfamily conotoxins belonging to the cysteine framework I [connectivity I–III, II–IV] show 40–88% identity to the known nAChR inhibitors (α-PIB, SrIA, SrIB, ArIA, ArIB, Ac1.1a, Ac1.1b, α-TxIA and TxIA(A10L)) belonging to the same gene superfamily with identical disulfide bridges and cysteine arrangement. Similarly, a sequence similarity search for PIVE and PIVF, A superfamily conotoxins belonging to cysteine framework IV [connectivity I–V, II–III, IV–VI] show 61–64% identity to the known nAChR inhibitors (OIVA and PeIVA). Both conotoxins also show 75% identity to the known NET/SLC6A2_inhibitor, Ar1311 (Figure 2). Norepinephrine transporter (NET) inhibitors have demonstrated efficacy in the treatment of children with attention-deficit hyperactivity disorder (ADHD) [72]. Additionally, the nAChR inhibitor Xen2174 (Mr1A) is in phase II clinical trials as a NET inhibitor tor the treatment of pain [73]. Thus PIVE and/or PIVF may have the potential to be developed into therapeutic drugs for the treatment of ADHD and/or pain.

**Figure 3:**
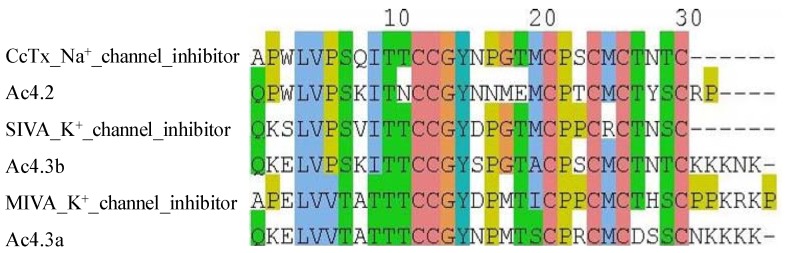
Multiple alignment of A superfamily conotoxins belonging to cysteine framework IV predicted to target both Na^+^ and K^+^ channels.

A superfamily conotoxins belonging to cysteine framework IV [connectivity I–V, II–III, IV–VI] (Ac4.2, Ac4.3a and Ac4.3b) also show 72–89% identity to known Na^+^ channel inhibitor, CcTx and 56–77% identity to K^+^ channel inhibitors, SIVA and MIVA. Thus, these conotoxins may be ideal candidates to increase understanding of interactions between the conotoxins and voltage- and ligand-gated ion channels (Figure 3). Other A superfamily conotoxins belonging to cysteine framework XIV [connectivity I–III, II–IV] show 78% (Ts14a) and 52–93% (As14b, As14a) identity to known K^+^ channel inhibitors Pu14a and vi11a, respectively. Sr11b and Sr11c, I2 superfamily conotoxins belonging to cysteine framework XI [connectivity I–IV, II–VI, III–VII, V–VIII] show 52–56% identity to K^+^ channel inhibitor, Sr11a and 43–52% identity to the calcium activated K^+^ channel inhibitor, BeTX (Supplementary Figure S1). 

The sequence similarity search for conotoxins belonging to the T superfamily with cysteine framework V (Pu5.1, Pu5.4, Pu5.5, Pu5.6, Vi1359, Vi1361, Sr5.4, Sr5.5, Sr5.6 and Sr5.7) showed 40–75% identity to known Na^+^ channel inhibitor, Lt5d. A member of the M superfamily with cysteine arrangement III, Pr3b and a member of the I1 superfamily with cysteine framework XI, R11d, also showed 55% and 98% identity to known Na^+^ channel inhibitors, PIIIA and Fi11.6, respectively (Supplementary Figure S1). Whilst, De7b and Pr6a, both members of the O1 superfamily with cysteine framework VI/VII [connectivity I–IV, II–V, III–VI] show 70 and 46% identity to Ca^2+^ channel inhibitors, TxO1 and PnVIA, respectively. PnVIA in particular have been demonstrated to block dihydropyridine-insensitive high voltage-activated calcium channels [74]. This calcium channel type has demonstrated sensitivity to nonselective T-type calcium channel antagonists and has been shown to contribute to the functioning of small cerebral arteries [75]. Thus, we suggest that De7b, Pr6a or analogs of these particular conotoxins may render more effective treatment for therapy-refractory cerebrovascular constriction.

## 4. Literature Analysis of Conotoxins Suggest Specific Therapeutic Potential

Curation of scientific literature draws attention to a similitude of neurodegenerative disorders (NDD) such as Alzheimer’s disease (AD), Parkinson’s disease (PD) and Multiple Sclerosis (MS) being characterized with aberrant neuronal excitability, caused by abnormal expression and function of ion channels. 

AD is characterized by neuronal loss of the superficial cortex and synaptic alterations such as reduction of pre-synaptic terminal density [76]. Mousavi *et al.* demonstrated that decreased nAChR subtypes α4β2 and α7 activities plays vital roles in the progression of AD [23,77,78]. This finding has been supported by recent studies that demonstrate that Aβ peptides can directly and indirectly affect nAChR-mediated synaptic transmission [79] and that nAChR agonists increase sAPPα secretion whilst decreasing levels of Aβ peptides [80]. Increased intracellular Ca^2+^ has also been implicated in the pathogenesis of AD. Specifically, Kim *et al.* demonstrated that Aβ increases the activities of L-type Ca^2+^ channel subtype Ca_v_1.2 and Ca_v_1.3 [81] and a calcium channel blocker was shown to ameliorate AD [82]. Ye *et al.* additionally showed that activation of the large-conductance Ca^2+^-activated K^+^ (BK) channel depresses the basal synaptic transmission in the hippocampal CA1 area in APP (swe/ind) TgCRND8 mice [83]. This demonstration of activated BK channels in AD may likely be attributed to the impaired calcium homeostasis. 

PD is characterized by a progressive loss of midbrain dopaminergic neurons and a subsequent reduction of striatal dopamine [84]. Perez *et al.* demonstrated that nAChR subtypes α4β2 and α6β2 are important modulators of dopaminergic transmission in the striatum and thus play a vital role in the progression of PD [85]. In addition, Kawamata *et al.* also demonstrated that nAChR subtypes α7 triggers multiple pathways that attenuate cytotoxicity in models of PD [86]. Ca^2+^ channels have also been implicated in the progression of PD, Tai *et al.* demonstrated that T-type Ca^2+^ channels are necessary for subthalamic burst firing and that pharmacological blockade of T-type Ca^2+^ channels reduces motor deficits in a rat model of PD [87]. Martel *et al.* further demonstrated that K_v_1.2, K_v_1.3 and K_v_1.6 are key regulators in Dopamine release, the dysfunction of which is thought to be implicated in PD [33]. Since SK channels have been demonstrated to play an important role in modulating synaptic plasticity, dopaminergic neurotransmission, and learning and memory, recent reviews have focused on the contradictory roles of SK channels in modulating dopaminergic neurons in substantia nigra and whether modulation of SK channels could be a potential target for PD treatment [88].

MS is characterized by focal destruction of myelin sheaths, gliotic scars, and axonal damage [89]. Craner *et al.* demonstrated that Na_v_1.2 and Na_v_1.6 are distributed along extensive regions of demyelinated axons within acute MS plaques and that Na_v_1.6 which can be driven by persistent sodium current to import damaging levels of calcium into axons, is colocalized with Aβ, a marker of axonal injury, in acute MS lesions [90]. Craner *et al.* further demonstrated the distribution of Na_v_1.6 in microglia and macrophages in experimental autoimmune encephalomyelitis (EAE) and MS and its key role in their activation and phagocytosis. Additionally, treatment with a sodium channel blocker was shown to ameliorate neuroinflammatory disorder via anti-inflammatory mechanisms [91]. Similarly, Brand-Schieber and Werner demonstrated increased expression of L-type Ca^2+^ channel subtype Ca_v_1.3 in mouse spinal cord axons and that calcium channel blockers ameliorated experimental autoimmune encephalomyelitis (EAE), an animal model of MS [92]. K^+^ channels have also been implicated in the pathogenesis of MS, as Wulff *et al.* demonstrated increased expression K^+^ channel subtype K_v_1.3 in activated myelin-reactive T cells from patients with MS [93]. 

**Figure 4:**
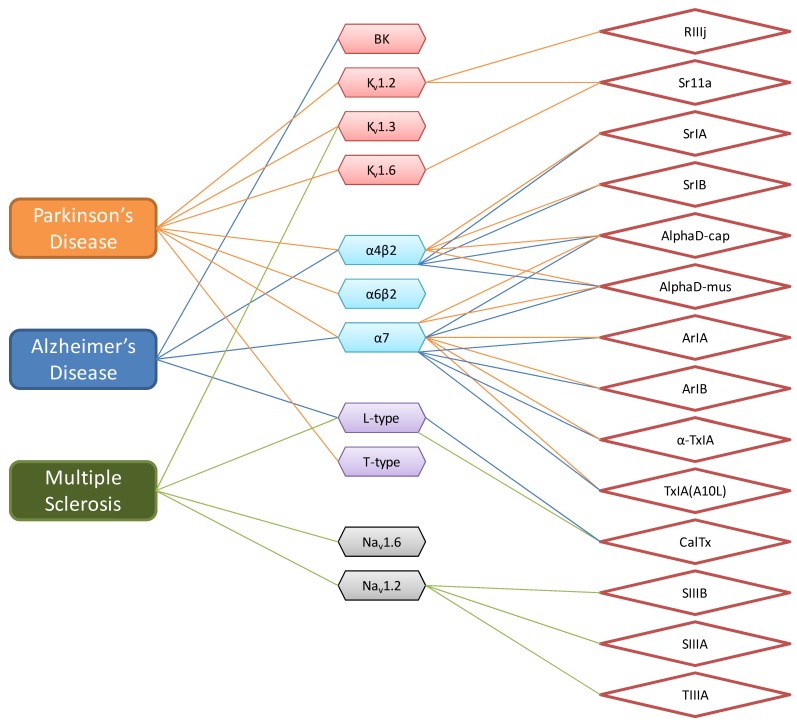
Schematic representation linking neurodegenerative disorders (NDD) to conotoxins with therapeutic potential.

Since research findings demonstrate that drugs capable of altering the abnormal expression and function of the membrane ion channels characterizing the individual disease states have therapeutic potential [94]. We curated the ion channels associated with the progression of the above mentioned NDD and associated the curated ion channels with the recently identified conotoxins that have been demonstrated to target these ion channels (NDD→COMMON ION CHANNEL→CONOTOXIN) (Figure 4).

The schema represents potential links of NDD to the recently identified conotoxins based on the fact that these conotoxins target one of the ion channels associated with the pathogenesis of the NDD. AD and PD have been linked to two nAChR inhibitors (αD-cap and αD-mus) with cysteine arrangement -C-CC-C-CC-C-C-C-C- of the D superfamily and six nAChR inhibitors (SrIA, SrIB, ArIA, ArIB, α-TxIA and TxIA(A10L)) with the cysteine arrangement -CC-C-C- from the A superfamily. PD has additionally been linked to two K^+^ channel inhibitors (Sr11a and RIIIj) with the cysteine arrangements -CC-CC-C-C- and -CC-C-C-CC- of the I2 and M superfamilies, respectively. Whilst MS has been linked to three Na^+^ channel inhibitors (SIIIA, SIIIB and TIIIA) with the cysteine arrangement -CC-C-C-CC- of the M superfamily. AD and MS has also been linked to Ca^2+^ channel inhibitor (CalTx) since it has been demonstrated that CalTx inhibits L-type Ca^2+^ channel. However, it must be noted that it had not been demonstrated which specific L-type Ca^2+^ channel subtype is inhibited by CalTx. In summary, the selected conotoxins or analogs thereof may possess therapeutic potential in treatment of NDD. 

## 5. Concluding Remarks

Although the possible application of conotoxins to treat NDD have not been researched as extensively as analgesic applications, current scientific literature produced illustrates that several diverse conotoxin families have demonstrable potential for the treatment of NDD and that conotoxins targeting both voltage-gated and ligand-gated ion channel families have potential in treatment of NDD. With respect to the important physiological role of voltage- and ligand-gated ion channels in pain, inflammation and disease states, targeting specific relevant voltage- and ligand-gated ion channel subtypes could be an attractive pharmaceutical strategy, with conotoxins as promising drug development leads. 

## Supplementary Files

Supplementary File 1: PDF-Document (PDF, 2369 KB)
