# Supplementary material for: Conotoxins that Confer Therapeutic Possibilities
Source: Mar Drugs. 2012 Jun 4;10(6):1244–65. doi: 10.3390/md10061244 (PMC3397437; doi:10.3390/md10061244)
Supplement: Supplementary File 1: — PDF-Document (PDF, 2369 KB) [file marinedrugs-10-01244-s001.pdf]

Supplementary Materials

Table S1. *Conus* peptides identified from 1 January 2007 to 31 August 2011.

**Peptide**	**Gene Superfamily and Cysteine framework**	**Type**	**Sequence**	**Predicted target**
Ts14a	A superfamily<br>XIV [connectivity I-III, II-IV]	α1-conotoxin	DGCPPHPVPGMHPCMCTNTC	nAChR
alpha-conotoxin	A superfamily<br>I [connectivity I-III, II-IV]	α-conotoxin	GGCCSHPACQNNPDYC	nAChR
Lp1.1	A superfamily<br>I [connectivity I-III, II-IV]	α-conotoxin	G**CC**ARAA**C**AGIHQEL**C**	nAChR
Lp1.10	A superfamily<br>I [connectivity I-III, II-IV]	α-conotoxin	ND**CC**HNAP**C**RNNHPGI**C**	nAChR
Lp1.4	A superfamily<br>I [connectivity I-III, II-IV]	α-conotoxin	G**CC**SHPA**C**SGNHQEL**C**D	nAChR
Lp1.6a	A superfamily<br>I [connectivity I-III, II-IV]	α-conotoxin	QF**CC**GHYD**C**DFIPNV**C**	nAChR
Lp1.6b	A superfamily<br>I [connectivity I-III, II-IV]	α-conotoxin	QI**CC**GYGD**C**GFVPNV**C**V	nAChR
Lp1.7	A superfamily<br>I [connectivity I-III, II-IV]	α-conotoxin	GMWDE**CC**DDPP**C**RQNNMEH**C**PAS	nAChR
Lp1.8	A superfamily<br>I [connectivity I-III, II-IV]	α-conotoxin	GVWDE**CC**KDPQ**C**RQNHMQH**C**PAR	nAChR
Lp1.9	A superfamily<br>I [connectivity I-III, II-IV]	α-conotoxin	**CC**SDSD**C**NANHPDM**C**S	nAChR
Mr1.1	A superfamily<br>I [connectivity I-III, II-IV]	α-conotoxin	G**CC**SHPA**C**SVNNPDI**C**	nAChR
Mr1.2	A superfamily<br>I [connectivity I-III, II-IV]	α-conotoxin	G**CC**SNPP**C**YANNQAY**C**N	nAChR
Mr1.3	A superfamily<br>I [connectivity I-III, II-IV]	α-conotoxin	G**CC**SHPA**C**RVHYPHV**C**Y	nAChR
Pu1.1	A superfamily<br>I [connectivity I-III, II-IV]	α-conotoxin	QN**CC**NVPG**C**WAKYKHL**C**	nAChR
Pu1.2	A superfamily<br>I [connectivity I-III, II-IV]	α-conotoxin	GG**CC**SYPP**C**IANNPL**C**	nAChR
Qc αL-1	A superfamily<br>I [connectivity I-III, II-IV]	α-conotoxin	TVRRFCSDPPCRISNPESCGW	nAChR
Qc αL-2	A superfamily<br>I [connectivity I-III, II-IV]	α-conotoxin	TVRGFCSDPSCRFGNPELCDW	nAChR
Qc1.1a	A superfamily<br>I [connectivity I-III, II-IV]	α-conotoxin	DE**CC**PDPP**C**KASNPDL**C**DWRS	nAChR
Qc1.1b	A superfamily<br>I [connectivity I-III, II-IV]	α-conotoxin	NE**CC**DNPP**C**KSSNPDL**C**DWRS	nAChR
Qc1.1c	A superfamily<br>I [connectivity I-III, II-IV]	α-conotoxin	DD**CC**PNPP**C**KASNPDL**C**DWRS	nAChR

Qc1.2	A superfamily<br>I [connectivity I-III, II-IV]	α-conotoxin	Q**CC**ANPP**C**KHVN**C**	nAChR
Qc1.4a	A superfamily<br>I [connectivity I-III, II-IV]	α-conotoxin	DG**CC**SNPS**C**SVNNPDI**C**	nAChR
Qc1.4b	A superfamily<br>I [connectivity I-III, II-IV]	α-conotoxin	DG**CC**PNPS**C**SVNNPDI**C**	nAChR
Qc1.5	A superfamily<br>I [connectivity I-III, II-IV]	α-conotoxin	G**CC**SNPA**C**SVNHPEL**C**	nAChR
Qc1.6	A superfamily<br>I [connectivity I-III, II-IV]	α-conotoxin	G**CC**SNPT**C**AGNNGNI**C**	nAChR
Ac4.2	A superfamily<br>IV [connectivity I-V, II-III, IV-VI]	κ-conotoxin	QPWLVPSKITN**CC**GYNNMEM**C**PT**C**M**C**T<br>YS**C**RP	K^+ channel
Ac4.3a	A superfamily<br>IV [connectivity I-V, II-III, IV-VI]	κ-conotoxin	QKELVVTATTT**CC**GYNPMTS**C**PR**C**M**C**DS<br>S**C**NKKKK	K^+ channel
Ac4.3b	A superfamily<br>IV [connectivity I-V, II-III, IV-VI]	κ-conotoxin	QKELVPSKITTCCGYSPGTACPSCMCTNT<br>CKKKNK	K^+ channel
Ar11a	I1 superfamily<br>XI [connectivity I-IV, II-VI, III-VII, V-VIII]	ι-conotoxin	RT**C**SRRGHR**C**IRDSQ**CC**GGM**CC**QGNR**C**<br>FVAIRR**C**FH	Na^+ channel
R11d	I1 superfamily<br>XI [connectivity I-IV, II-VI, III-VII, V-VIII]	ι-conotoxin	GCKKDRKPCSYHADCCNCCLSGICAPSTN<br>WILPGCSTSTFT	Na^+ channel
Eb12.4	I2 superfamily<br><u>XII</u>	E-conotoxin	S**C**DSEFSSEF**C**EQPEERI**C**S**C**STHV**CC**HLSS<br>SKRDQ**C**MTWN<br>R**C**LSAQTGN	ND
Im12.10	I2 superfamily<br><u>XII</u>	E-conotoxin	L**C**DSYISSEL**C**EHPEET**C**F**C**PNHM**CC**PLSP<br>YRQDQ**C**MYWEA<br>**C**HIF	ND
Lt12.4	I2 superfamily<br><u>XII</u>	E-conotoxin	W**C**SERVSDET**C**VMM**C**R**C**LNHE**CC**PLPP<br>PSQNR**C**MPSDH**C**DF<br>MSGRT	ND
Lt12.9	I2 superfamily<br><u>XII</u>	E-conotoxin	S**C**GVRISSEI**C**EQPEERI**C**S**C**SNHM**CC**PLN<br>PSQRDQ**C**MARN<br>V**C**FIMIGIYG	ND
Mr12.5	I2 superfamily<br><u>XII</u>	E-conotoxin	RI**C**S**C**STHV**CC**HLSSSKRDQ**C**MTWNR**C**L<br>SAQTGN	ND
Mr12.8	I2 superfamily<br><u>XII</u>	E-conotoxin	S**C**DSEFSSEF**C**EQPEERI**C**S**C**STHV**CC**HLSS<br>SKGDQ**C**MTWN<br>R**C**LSAQTGN	ND
Sr11b	I2 superfamily<br>XI [connectivity I-IV, II-VI, III-VII, V-VIII]	κ-conotoxin	**C**DSDGTS**C**TSNME**CC**GYG**CC**SGT**C**QTP**C**<br>RFGP	K^+ channel
Sr11c	I2 superfamily<br>XI [connectivity I-IV, II-VI, III-VII, V-VIII]	κ-conotoxin	**C**SDEGAS**C**EKKSD**CC**FLS**CC**WSV**C**DRP**C**R<br>LVP	K^+ channel

Ca11a	I3 superfamily<br>XI [connectivity I-IV, II-VI, III-VII, V-VIII]	I-conotoxin	AWP**C**GGVRAS**C**SRHDD**CC**GSL**CC**FGTST G**C**RVAVRP**C**W	
Ca11b	I3 superfamily<br>XI [connectivity I-IV, II-VI, III-VII, V-VIII]	I-conotoxin	ALL**C**GGTHAR**C**NRDND**CC**GSL**CC**FGT**C**IS AFVP**C**	
Pr3a	III [connectivity I-IV, II-V, III-VI]	μ-conotoxin	**CC**NWP**C**SFG**C**IP**CC**Y	Na^+ channel
Pr3b	III [connectivity I-IV, II-V, III-VI]	μ-conotoxin	ERV**CC**GYOMS**C**KSRA**C**KOSY**CC**	Na^+ channel
PIVE	IV [connectivity I-V, II-III, IV-VI]	κ-conotoxin	D**CC**GVKLEM**C**HP**C**L**C**DNS**C**KNYGK	ND
PIVF	IV [connectivity I-V, II-III, IV-VI]	κ-conotoxin	D**CC**GVKLEM**C**HP**C**L**C**DNS**C**KKSGK	ND
Sr7a	O1 superfamily<br>VI/VII [connectivity I-IV, II-V, III-VI]	conotoxin	**C**LQFGST**C**FLGDDDI**CC**SGE**C**FYSGGTFGI **C**S	ND
Ca8a	S superfamily<br>VIII	conotoxin	G**C**SGT**C**HRREDGK**C**RGT**C**D**C**SGYSY**C**R**C** GDAHHFYRG**C**T**C**T**C**	ND
Pu5.1	T superfamily<br>V [connectivity I-III, II-IV]	T-1 conotoxin	S**CC**PSPTS**CC**PW	ND
Pu5.2	T superfamily<br>V [connectivity I-III, II-IV]	T-1 conotoxin	G**CC**EDKT**CC**FI	ND
Pu5.3	T superfamily<br>V [connectivity I-III, II-IV]	T-1 conotoxin	S**CC**PEEP**CC**FW	ND
Pu5.4	T superfamily<br>V [connectivity I-III, II-IV]	T-1 conotoxin	S**CC**PEEIT**CC**PW	ND
Pu5.5	T superfamily<br>V [connectivity I-III, II-IV]	T-1 conotoxin	E**CC**PQSPP**CC**HYYYYGSW	ND
Pu5.6	T superfamily<br>V [connectivity I-III, II-IV]	T-1 conotoxin	**CC**PRKIW**CC**MIP	ND
Vi1359	T superfamily<br>V [connectivity I-III, II-IV]	T-1 conotoxin	Z**CC**ITIPE**CC**RI	ND
Vi1361	T superfamily<br>V [connectivity I-III, II-IV]	T-1 conotoxin	Z**CC**PTMPE**CC**RI	ND
Sr5.4	T superfamily<br>V [connectivity I-III, II-IV]	T-1-conotoxins	IINW**CC**LVFYQ**CC**	ND
Sr5.5	T superfamily<br>V [connectivity I-III, II-IV]	T-1-conotoxins	IINW**CC**LTFYQ**CC**	ND
Sr5.6	T superfamily<br>V [connectivity I-III, II-IV]	T-1-conotoxins	IMAGCCPRFYQCCYP	ND
Sr5.7	T superfamily<br>V [connectivity I-III, II-IV]	T-1-conotoxins	IINW**CC**LIFYQ**CC**L	ND
Vi15a	V superfamily<br>XV	V-conotoxin	D**C**TT**C**AGEE**CC**GR**C**T**C**PWGDN**C**S**C**IEW	5-HT_3 receptor
Pr6a	VI/VII [connectivity I-IV, II-V, III-VI]	conotoxin	T**C**LARDEL**C**GASFLSNFL**CC**DGL**C**LLI**C**V	ND
Pr6b	VI/VII [connectivity I-IV,	conotoxin	FGSFIO**C**AHKGEO**C**TI**CC**ROLR**C**HEEKTO	ND

	II-V, III-VI]		T**C**V	
Pr6c	VI/VII [connectivity I-IV, II-V, III-VI]	conotoxin	DQ**C**TY**C**GIY**CC**PPKF**C**TSSG**C**RSP	ND
Pr6d	VI/VII [connectivity I-IV, II-V, III-VI]	conotoxin	YGNFOT**C**SETGED**C**SAMH**CC**RSMT**C**RN NI**C**AD	ND
De7b	VI/VII [connectivity I-IV, II-V, III-VI]	δ-conotoxin	DCIPGGENCDVFRPYRCCSGYCILLLCA	Na^+ and/or Ca^{2+} channel
Mr1e	X [connectivity I-IV, II-III]	α-conotoxin	**CC**HSSW**C**KHL**C**	nAChR
As14a	XIV [connectivity I-III, II-IV]	κ-conotoxin	GGVGR**C**IYN**C**MNSGGGLNFIQ**C**KTM**C**Y	K^+ channel
As14b	XIV [connectivity I-III, II-IV]	κ-conotoxin	WDVDQ**C**IYY**C**LNGVVGYSYTE**C**QTM**C**T	K^+ channel
Qc16a	XVI	conotoxin	D**C**QP**C**GHNV**CC**	
P21a	XXI	conotoxin	S**CC**IQKTLE**C**LENYOGQASQRAHY**C**QQD ATTN**C**ODTYYFG**CC**PGYAT**C**MSINAGNV RSAFDK**C**INRL**C**FDPGH	AMPA receptor
Ca16a	Y superfamily XVII	conotoxin	**C**GGTGDS**C**NEOAGEL**CC**RRLK**C**VNSR**CC** PTTDG**C**	ND

Figure S1. Multiple alignments of predicted conotoxin targets.

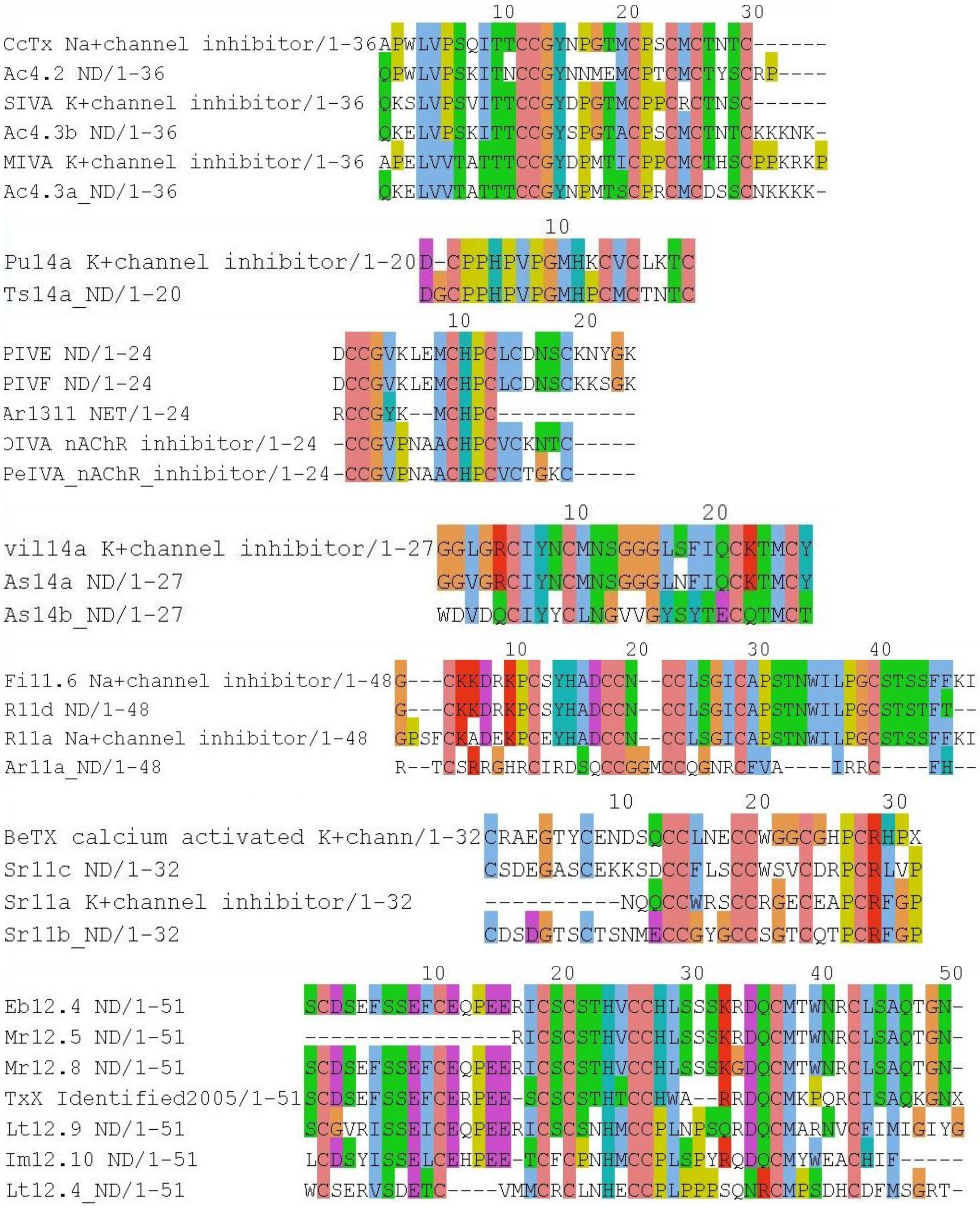

```
                                                 10
CalTx Ca2+channel inhibitor/1-13NCPAGCRSQGCCM
Qc16a_ND/1-13                   DCQP-CGHNVCC-

                                       10        20        30        40        50        60        70        80
Con-ikot-ikot AMPA/1-87SGPADCCRMKECCTDRVNECLQRYSGREDKFVSFCYQEATVTC-GSFNEIVGCCYGYQMCMIRVVKPNSLSGAHEACKTVSCGNPCA
P21a_ND/1-87           ----------SCCIQKTLECLENYOGQASQRAHYCQQDATTNCODTY--YFGCCPGYATCMS--INAGNVRSAFDKCINRLCFDPGH

                                                    10        20        30        40
Mu-hexatoxin-Mg2a Na+channel i/1-46GGCIKWNHSCQTTTLKCCGKCVVCYCHTPWGTNCRCDRTRLFCTED
Omega-plectoxin-Pt1a Ca2+chann/1-46ADCSATGDTCDHTK-KCCDDCYTCRCGTPWGANCRCDYYKARCDT-
Vi15a_ND/1-46                      -DCT----TCAGEE--CCGRC-TC-----PWGDNCSCIEW-------

                                          10        20
RIIIj K+channel inhibitor/1-29   -LPPCCTP--PKKHCPAPACKYKP-CCKS
PrIIIE nAChR inhibitor/1-29      -AARCCT---YHGSCLKEKCRRKY-CCGR
PIIIA Na+channel inhibitor/1-29  -QRLCCG---FPKSCRSRQCKPHR-CC--
Pr3b ND/1-29                     -ERVCCG---YOMSCKSRACKOSY-CC--
BuIIIA Na+channel inhibitor/1-29VTDRCCK---GKRECG-RWCRDHSRCC--
BuIIIB Na+channel inhibitor/1-29VGERCCKN--GKRGCG-RWCRDHSRCC--
BuIIIC Na+channel inhibitor/1-29IVDRCCNKGNGKRGCS-RWCRDHSRCC--
SIIIA Na+channel inhibitor/1-29  --ZNCCN-----GGCSSKWCRDHARCC--
SIIIB Na+channel inhibitor/1-29  --ZNCCN-----GGCSSKWCKGHARCC--
TIIIA Na+channel inhibitor/1-29  -RHGCCKGO---KGCSSRECRPQH-CC--
                                        10        20        30
TxO1 Ca2+channel inhibitor/1-32  -CLDAGEVCDIFFP---TCC-GYCILLFCA--
De7b ND/1-32                      DCIPGGENCDVFRPY--RCCSGYCILLLCA--
PnVIA Ca2+channel inhibitor/1-32GCLEVDYFCGIPFANNGLCCSGNCVFV-CTPQ
Pr6a_ND/1-32                      TCLARDELCGASFLSNFLCCDGLCLLI-CV--
                                          10
Lt5d Na+channel inhibitor/1-16---DCCPAKLLCCN-P
Pu5.6 ND/1-16                  ----CCPRKIWCCMIP
Vi1359 ND/1-16                 ---ZCCITIPECCRI-
Vi1361 ND/1-16                 ---ZCCPTMPECCRI-
Pu5.3 ND/1-16                  ---SCCPEE-PCCFW-
Pu5.4 ND/1-16                  ---SCCPEEITCCPW-
Pu5.1 ND/1-16                  ---SCCPSPTSCCPW-
Pu5.2 ND/1-16                  ---GCCEDK-TCCFI-
Sr5.4 ND/1-16                  IINWCCLVFYQCC---
Sr5.7 ND/1-16                  IINWCCLIFYQCCL--
Sr5.5 ND/1-16                  IINWCCLTFYQCC---
Sr5.6_ND/1-16                  IMAGCCPRFYQCCYP-
```
